# A comparison of the level of functioning in chronic schizophrenia with coping and burden in caregivers

**DOI:** 10.4103/0019-5545.31615

**Published:** 2006

**Authors:** Dean A. Creado, Shubhangi R. Parkar, Ravindra M. Kamath

**Affiliations:** * Former Postgraduate Resident; *** Former Associate Professor; ** Professor and Head

**Keywords:** Chronic schizophrenia, level of functioning, caregivers, mechanisms of coping, burden of illness

## Abstract

**Background:**

A chronic mental illness such as schizophrenia is a challenging task for caregivers especially in the current era of de-institutionalization. In India, few studies have attempted to directly determine the relationship between coping mechanisms and burden; in the West, studies have found that improved coping in family members can decrease the perceived burden.

**Aim:**

To evaluate the burden and coping of caregivers in relation to the level of functioning in patients with chronic schizophrenia.

**Methods:**

The sample was 100 patients with their primary caregivers attending a Psychiatry OPD. Patients were assessed on the Global Assessment of Functioning (GAF) scale while caregivers were administered the Burden Assessment Schedule (BAS) and Mechanisms of Coping (MOC) scale.

**Results:**

Fatalism and problem-solving were the two most preferred ways of coping. Problem-focused coping, i.e. problem-solving and expressive-action decreased the burden of caregivers, while emotion-focused coping, i.e. fatalism and passivity, increased it. As the level of functioning of the patient decreased, the significance with which the coping mechanisms influenced the burden, increased. The use of problem-solving coping by caregivers showed a significant correlation with higher level of functioning in patients.

**Conclusion:**

Coping mechanisms such as problem-solving can decrease the burden of illness on caregivers and may even improve the level of functioning of patients.

## INTRODUCTION

Schizophrenia is the paradigmatic illness of psychiatry. The policy of de-institutionalization has highlighted the role of family members as the primary source of caregiving for relatives with schizophrenia.^1^ A noteworthy finding by Weidmann *et al.* was that despite the apparent downfall of traditional family structure, over *60%* of patients with long-term schizophrenia live with at least one ‘significant other’, i.e. primary caregiver.^2^

The demands of caring for a mentally ill relative have both an emotional and practical impact on the caregiver,^3^,^4^ which have been defined and quantified by concepts of subjective and objective burden.^5^,^6^ The fact that the illness leaves a varying degree of disability in the patient^7^ and leads to disturbing behaviour means that its management is associated with a significant burden of care.^3^,^8^

However, not all caregivers perceive the same burden of illness because it varies according to their ways of coping. Folkman and Lazarus have defined coping as a person's constantly changing cognitive and behavioural efforts to manage an encounter appraised as stressful.^9^ Birchwood and Cochrane found that relatives of patients with schizophrenia employed a broad range of coping styles in response to behavioural changes in patients.^10^ Both emotion-focused and problem-focused coping lead to reappraisal of the stressful event, i.e. patients' illness.^11^

The relationship between coping styles and perceived burden of care is complex because caregivers subjectively report ‘burden’. This subjectivity in turn is a product of the coping styles used by caregivers. In 1994, the consensus reported by Troop states that emotion-based coping is associated with an unsatisfactory outcome whereas problem-focused coping is associated with a more satisfactory outcome.^12^ These findings suggest that the burden of care givers is more dependent on their appraisal of the condition of their patients rather than the actual illness.^13^,^14^

Pai and Kapur observed that in view of the economic and cultural conditions of a developing country being vastly different from those of the western world, the areas of burden and the pattern of accepting or rejecting patients in India may be entirely different.^15^ Wig *et al.* in 1987 also found that expressed emotion as a concept associated with burden plays a relatively less significant role in families.^16^ Not many studies have examined the ways in which relatives cope while caring for a patient with schizophrenia and the relationship of coping styles to burden. Thus, it is more relevant to study the burden of caregivers and their adjustment to the level of functioning of patients as shown by various coping strategies employed by caregivers.^17^

In the words of Helene Provencher: ‘Although behavioural disturbances in schizophrenia have been studied for more than two decades, additional work is needed to better understand the caregivers perspective.’^4^ Indeed, the influence of coping styles on burden experienced by caregivers would help us evaluate and plan effective programmes that address their needs and teach them adaptive mechanisms of coping. This would enable them to focus on the positive feelings they experience in association with the caregiving role and ways to sustain this positive well-being.

## AIMS

The present study was conducted on primary caregivers of patients with chronic schizophrenia living in the community. The study aimed to assess:

1. The level of functioning in patients with chronic schizophrenia.
2. The burden of illness and coping mechanisms of the primary caregiver.
3. The relation between the level of functioning in patients, the coping styles used by caregivers and their perceived burden of illness.

## METHODS

The approval for the study was obtained from the institution's Ethics Committee.

*Study site:* The follow-up Psychiatry OPD of an urban municipal general hospital.

*Study design:* Clinical, instrument-rated and cross-sectional.

*Sample selection: 100* primary caregivers regularly accompanying patients diagnosed as having chronic schizophrenia as per the DSM criteria^18^ and who attend the follow-up OPD.

### Inclusion criteria

A random selection was made, if the following inclusion criteria were satisfied.

#### For patients

- >18 years old
- Diagnosis of chronic schizophrenia as per the DSM criteria
- On regular follow up for the past 6 months and on medication.

#### For caregivers

- 18 years old
- The primary caregiver was identified as an adult relative living with a patient, in the same environment, for at least 12 months and was involved directly in giving care to the patient and most supportive either emotionally or financially, i.e. felt most responsible for the patient.^19^,^20^

Patients with an exacerbation of symptoms in the past 6 months or any documented psychiatric co-morbidity as per the DSM criteria, except nicotine dependence, were excluded. We also did not include patients or caregivers who had any incapacitating medical illness or language incompatibility.

*Data collection:* Over a period of 6 months, patients and corresponding caregivers who satisfied the criteria were interviewed after obtaining their written informed consent. The data were recorded and further aspects were studied as described below.

*Tools:* These consisted of a semi-structured interview covering the sociodemographic profile, details of illness and functioning of the patients as defined by the DSM criteria^18^ for schizophrenia with its various subtypes and three scales.

1. The Global Assessment of Functioning (GAF) scale is a measure of rating the overall psychological, social and occupational functioning of the patient, first included in DSM-III-R as axis-V of the multi-axial diagnostic system. It is a modified version of ‘The Global Assessment Scale: A procedure for measuring overall severity of psychiatric disturbances’ developed by Endicott *et al.* in 1976^21^. The scale has 10 ranges of functioning where each range has two components covering symptom severity and patient functioning. The final GAF rating always reflects the worse of the two. It excludes impairment due to physical or environmental limitations.^18^
2. The Burden Assessment Schedule (BAS) developed by Thara *et al.*^22^ at the Schizophrenia Research Foundation (SCARF) is based on the principle of ‘stepwise ethno-graphic exploration’ described by Sell and Nagpal in 1992 while studying affected families in an effort to gauge the ‘meaning’ of giving care to a chronic psychotic person.^23^ This is a semi-quantitative, 40-item scale measuring 9 different areas of objective and subjective caregiver burden. Each item is rated on a 3-point scale. Scores range from 40 to 120 with higher scores indicating greater burden. Internal consistency for the full scale is 0.80 as measured by the alpha coefficient.^11^ Its validity is comparable with the Family Burden Schedule (FBS) by Pai and Kapur.^15^
3. The Mechanisms of Coping (MOC) scale was modified by Parikh *et al.*^24^ from the ‘Ways of Coping Scale’ by Lazarus and Folkman.^11^ It is a 30-item instrument, which contains 24 select items of the original scale as well as 6 new items related to fatalism. The 30 items are divided into 5 factors that relate to individual ways of coping; escape-avoidance, fatalism, expressive-action, problem-solving and passivity.

Scores range from 0—‘not used at all’ to 3—‘used a great deal’. Average score for each factor was calculated by dividing the total score by the number of items comprising that factor to get the mean factor score. This was used to derive the relative factor score across 100 subjects. A higher relative factor score on a scale indicates that the caregiver used that particular coping mechanism more often. This scale correlates well with the original 65-item scale in assessment of coping styles.

*Statistical analysis:* The sample was divided into two groups using the distribution of patients by their level of functioning. Using SPSS-10 for Windows, analysis of the relationship between variables such as the level of functioning in the patient, caregiver coping mechanisms and burden of illness was studied with Pearson chi-square test, independent t test and Pearson correlation coefficient for statistical significance. A p value of <0.05 was taken as significant.

## RESULTS AND DISCUSSION

### Global assessment of functioning

**Fig 1:**
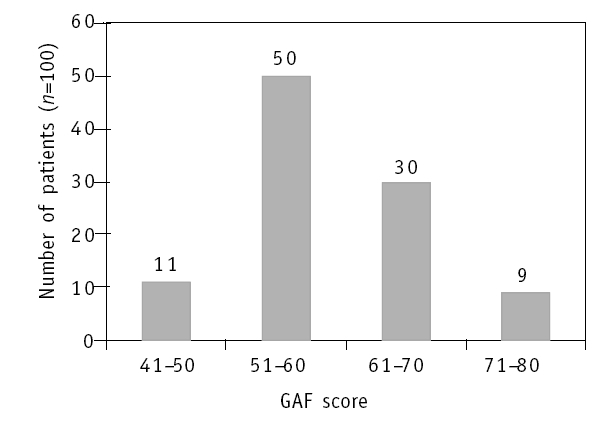
Patient distribution by the Global Assessment of Functioning (GAF) score

In this study, the scores of the patients were distributed in four ranges; from 41-50 to 71-80 (Fig 1). On combining, the scores showed that

- 61% of the patients had moderate to serious problems in functioning or symptoms with a GAF score of 41-60 (group A), while
- 39% had transient to mild problems in functioning or symptoms with a GAF score of 61-80 (group B).

These were taken as comparison groups. This finding is in keeping with that cited in the literature, which says that even with treatment, 20%-30% of patients continue to experience moderate symptoms and 40%-60% of patients remain significantly impaired throughout their lives.^25^

### Sociodemographic profile

This was compared for groups A and B. The variables were divided into patients' characteristics, socioeconomic factors and caregivers' characteristics.

#### Patients' characteristics vs functioning

Average age of the patients was around 36 years. Male:Female ratio was 1.56:1. Half the patients were still married and about two-thirds had nuclear families; 66% were suffering from schizophrenia for at least 10 years (Table 1).

**Table 1:** Sociodemographic profile: Patients' characteristics vs functioning

Patient variable	Variable subgroup	Level of functioning		Significance
			
		Group A *n*=61 (%)	Group B *n*=39 (%)	
Age	≤35 years	31 (50.8)	18 (46.2)	χ^2^=0.07
	≥36 years	30 (49.2)	21 (53.8)	df=1; p=0.8
Sex	Male	32 (52.5)	15 (38.5)	χ^2^= 1.87
	Female	29 (47.5)	24 (61.5)	df=1; p=0.17
Religion	Hindu	41 (67.2)	33 (84.6)	χ^2^=4.28
	Muslim	7 (11.5)	2 (5.1)	df=2; p=0.23
	Others	13 (21.3)	4 (10.3)	
Marital status	Never married	21 (34.4)	12 (30.8)	χ^2^= 0.19
	Married	32 (52.5)	21 (53.8)	df=2; p=0.11
	Previously married	8 (13.1)	6 (15.4)	
Reproductivity (i.e. children)	No	28 (45.9)	17 (43.6)	χ^2^= 0.05
	Yes	33 (54.1)	22 (56.4)	df=1; p=0.82
Rural/urban setting	Urban	47 (77)	35 (89.7)	χ^2^= 2.59
	Rural	14 (23)	4 (10.3)	df=1; p=0.11
Family	Nuclear	35 (57.4)	26 (66.7)	χ^2^=0.86
structure	Joint	26 (42.6)	13 (33.3)	df=1; p=0.35

*Chi-square test. Significant value of Pearson ‘p’<0.05

#### Socioeconomic factors vs functioning

This was compared for the patients in both groups—58% of patients had received some schooling; 42% were unemployed; and more than 60% were economically backward (Table 2).

**Table 2:** Socioeconomic factors vs functioning

Patient variable	Variable subgroup	Level of functioning		Significance
			
		Group A *n*=61 (%)	Group B *n*=39 (%)	
Education	0-IV std	18 (29.5)	6 (15.4)	χ^2^=2.622
	V-X std	28 (45.9)	22 (56.4)	df=2; p=0.270
	>X std	15 (24.6)	11 (28.2)	
Occupation	Unemployed	40 (65.6)	18 (46.2)	χ^2^=2.929
	Employed	21 (34.4)	21 (53.8)	df=1; p=0.087
Household	<5000	48 (78.7)	24 (61.5)	χ^2^=3.471
income (Rs/month)	>5000	13 (21.3)	15 (38.5)	df=1; p=0.062
Housing	*Kuccha* (street, slum)	11 (18)	10 (25.6)	χ^2^=0.830
				df=1; p=0.362
	*Pucca* (flat, chawl, etc.)	50.2	29 (74.4)	
House ownership	Owned	46 (75.4)	29 (74.4)	χ^2^= 0.14
	Rented	15 (24.6)	10 (25.6)	df=1; p=0.90
Spouse job	Unemployed	38 (62.3)	23 (58.9)	χ^2^=0.001
	Employed	20 (32.8)	12 (30.8)	df=1; p=0.92

*Chi-square test. Significant value of Pearson ‘p’<0.05

#### Caregivers’ characteristics vs functioning

Among caregivers, about two-thirds were either parents or spouses, over 35 years old. As expected, 61% were women and approximately 50% of the caregivers were unemployed (Table 3).

**Table 3:** Caregiver characteristics vs functioning

Caregiver variables	GAF score		Significance
		
	Group A (%)	Group B (%)	
*Age*			
≤35 years	15 (24.6)	10 (25.6)	χ^2^=0.01
≥36 years	46 (75.4)	29 (74.4)	df=1; p=0.90
*Sex*			
Male	25 (41)	14 (35.9)	χ^2^=0.26
Female	36 (59)	25 (64.1)	df=1; p=0.61
*Employment*			
No	29 (47.5)	17 (43.6)	χ^2^=0.15
Yes	32 (52.4)	22 (56.4)	df=1; p=0.69
*Caregiver role*			
Parent	26 (42.6)	17 (43.6)	χ^2^=1.240
Spouse	17 (27.9)	14 (35.9)	df=2; p=0.538
Others	18 (29.5)	8 (20.5)	
*Education*			
0-IV std	22 (36.1)	16 (41)	χ^2^=0.319
V-CX std	30 (49.2)	17 (43.6)	df=2; p=0.853
>X std	9 (14.7)	6 (15.4)	

*Chi-square test. Significant value of Pearson ‘p’<0.05

The data were first categorized and analysed using the chi-square (χ^2^) test. This was to see if the 2 groups matched so that further analysis would then be possible to determine the relationship between the level of functioning in patients, the various coping mechanisms used by caregivers and subse-quently the burden of illness felt by them.

Overall, groups A and B were matched for their socio-demographic profiles. This was in keeping with the population from which the sample was drawn. This means that these factors did not significantly affect the level of functioning in chronic schizophrenia.

### Level of functioning and burden

Using the Burden Assessment Schedule (BAS), the total burden scores were calculated as ‘Mean±SD’ and were significantly higher (p<0.001) in group A (78.52±12.70) than in group B (68.13±11.37) when compared with the unpaired *t* test (Table 4). Based on the total scores of burden of illness, the caregivers were divided into those with scores of >80 as a lower burden and >80 as experiencing a higher burden.

**Table 4:** Level of functioning in patients vs burden of caregivers

GAF	BAS^a^	No. of caregivers *n*(%)	Significance*
Group A	<80	24 (40)	χ^2^= 10.03; df=1;
(*n*=61)	>80	37 (60)	p=0.001* (highly significant)
Group B	<80	28 (72)	
(*n*=39)	>80	11 (28)	

* Chi-square test. Significant value of Pearson ‘p’<0.05; highly significant value ‘p’<0.01.

a Based on the total scores of burden of illness (BAS), the caregivers were divided into those who had a score of <80 as experiencing a lower burden and >80 as experiencing a higher burden.

In group A, despite patients with symptoms or impairment in functioning which was moderate to severe, 40% of caregivers reported a lower burden while in group B, despite patients with symptoms or impairment in functioning which was transient to mild, 28% of caregivers felt a higher burden.

The difference was statistically significant. This implied that in an illness such as schizophrenia, characterized by multiple exacerbations and remissions, not all caregivers perceive the same amount of burden.

This finding is in keeping with studies which suggest that burden of care is more dependant on caregivers' appraisal of the condition of patients than on the actual deficits and symptomatology of patients.^13^,^14^

### Mechanisms of coping

From the relative scores of coping effort by coping style, it was found that fatalism and problem-solving contributed 26.4% and 27.4% of the coping effort of caregivers respectively, followed by passivity at 22.4%, expressive-action at 13% and escape-avoidance at 10.8%. This is in keeping with the results of Birchwood and Cochrane^10^ who found that relatives use a broad range of coping styles.

Fatalism and problem-solving were the two most used mechanisms of coping. Generally, as Indians, we are fatalistic and usually look for divine intervention in a crisis. Even in a different cultural context such as an urban population, as reported by Parikh *et al.,* factors such as faith, myth and hope play a major role in the process of psychotherapy.^24^

This is partly in keeping with the results of Scazufca and Kuipers^20^ who reported that relatives used more problem-focused coping strategies. This finding is also reflected in the results of Folkman *et al.,*^26^ Magliano *et al.*^27^ and Chandrasekharan *et al.*^17^ who found that caregivers use emotion-focused coping such as resignation when they perceive the illness to be protracted and unchangeable.

**Table 5:** Relationship between patient's level of functioning, caregiver's coping styles and burden of illness: Correlation of BAS, GAF and coping mechanisms

Mechanism of coping	MOC Es-Av	MOC Fata	MOC Ex-Ac	MOC Pr-So	MOC Pas	GAF score	BAS score
1. Escape avoidance	**1.00**	0.117	−0.105	−0.135	0.303	−0.097	0.194
		p=0.12	p=0.15	p=0.09	p=0.0014*	p=0.17	p=0.027*
2. Fatalism	0.117	**1.00**	−0.526	−0.738	0.106	−0.149	0.435
	p=0.12		p=0.000*	p=0.000*	p=0.15	p=0.07	p=0.000*
3. Expressive action	−0.105	−0.526	**1.00**	0.247	−0.408	0.016	−0.354
	p=0.15	p=0.000*		p=0.009*	p=0.000*	p=0.44	p=0.000*
4. Problem-solving	−0.135	−0.738	0.247	**1.00**	−0.401	0.210	−0.531
	p=0.09	p=0.000*	p=0.009*		p=0.000*	p=0.022*	p=0.000*
5. Passivity	0.303	0.106	−0.408	−0.401	**1.00**	−0.006	0.264
	p=0.0014*	p=0.15	p=0.000*	p=0.000*		p=0.47	p=0.004*
GAF score	−0.097	−0.149	0.016	0.210	−0.006	**1.00**	−0.317
	p=0.17	p=0.07	p=0.44	p=0.022*	p=0.47	p=0.001*
BAS score	0.194	0.435	−0.354	−0.531	0.264	−0.317	**1.00**
	p=0.027*	p=0.000*	p=0.000*	p=0.000*	p=0.004*	p=0.001*	

*Pearson correlation coefficient (r); Significant value ‘p’<0.05; highly significant value ‘p’<p.01

### Relationship between patients’ level of functioning and caregivers’ coping styles and burden of illness

#### Correlation of scores on BAS and GAF with coping mechanisms

Analysis of the mechanisms of coping using the Pearson correlation coefficient showed that there was a highly significant positive correlation between escape-avoidance and passivity, both emotion-focused styles of coping. There was a highly significant positive correlation noted between expressive-action and problem-solving, i.e. all problem-focused styles of coping. Further, a significant negative correlation between the 2 groups of emotion-focused and problem-focused mechanisms of coping. There was a complex interplay between coping mechanisms, patient functioning and burden in caregivers as follows:

- Emotion-focused coping mechanisms of escape-avoidance, fatalism and passivity correlated significantly with higher burden.
- Problem-focused coping mechanisms such as expressive-action and problem-solving showed a significant negative correlation with burden.
- A singular result of a highly significant positive correlation was noted between the use of problem-solving coping style and the level of functioning in patients.

#### Coping mechanisms vs burden-comparison between groups A and B

The data were further analysed with the unpaired t test to examine these factors more closely (Table 6). The significant results here were similar for the following.

**Table 6:** Coping versus burden (comparison between groups)

Level of functioning	Group A (GAF: 41-60) (*n*=61)			Group B (GAF: 61-80) (*n*=39)		
		
Burden of illness (Total score)	<80	>80	Unpaired *t* test	<80	>80	Unpaired *t* test
				
Coping mechanism	MOCS relative score (Mean±SD)		Significance	MOCS relative scores (Mean±SD)		Significance
Escape-avoidance	0.09±0.05	0.12±0.07	*t*=1.24; p=0.22	0.09±0.04	0.12±0.10	*t*=1.21; p=0.23
Fatalism	0.20±0.11	0.31±0.10	*t*=3.93; p<0.001*	0.20±0.13	0.30±0.10	*t*=2.25; p=0.03*
Expressive-action	0.17±0.08	0.10±0.05	*t*=3.54; p=0.0011*	0.15±0.09	0.09±0.06	*t*=1.89; p=0.87
Problem-solving	0.34±0.12	0.22±0.06	*t*=5.21; p<0.001*	0.34±0.11	0.24±0.10	*t*=2.69; p=0.012*
Passivity	0.19±0.07	0.25±0.09	*t*=2.64; p=0.013*	0.21±0.08	0.24±0.09	*t*=1.13; p=0.26

* Unpaired *t* test; significant value ‘p’ <0.05; highly significant value ‘p’ <0.01

In group B, when caring for patients with transient to mild symptoms or impairment in functioning, those who reported a higher burden (>80) were using emotion-focused mechanisms of coping such as fatalism to a greater extent while caregivers with a lower burden score (<80) were using more problem-focused mechanisms of coping such as problem-solving.

In group A, when caring for patients with moderate to severe symptoms or impairment in functioning, use of emotion-focused mechanisms of coping such as fatalism and passivity to a greater extent was associated with a higher burden whereas caregivers who reported a lower burden were using more problem-focused mechanisms of coping such as problem-solving and expressive-action.

Thus, in patients who had transient to mild impairment in functioning, the burden experienced by caregivers did not vary much with the coping mechanisms used. However, in patients who had moderate to severe impairment in functioning, the burden was reduced significantly with the use of adaptive problem-focused coping mechanisms of expressive-action and problem-solving.

These findings are similar to those which suggest that burden of care is more dependent on the caregivers' appraisal of the situation.^13^,^14^ The findings of this study are not in complete agreement with Budd *et al.* who showed that the avoidance of maladaptive coping styles *per se* rather than the use of adaptive coping styles relates to decreased caregiver burden.^28^ The stress-coping theory of Lazarus and Folkman^11^ provides an intra-individual perspective of stress and explains how the reappraisal of patients' behaviour reshapes the coping situation into a more satisfying experience.^29^,^30^

This study also found that there was a significant positive correlation between the use of problem-solving coping and the level of functioning. This is a corollary to the finding of Chandrasekharan *et al.* who showed that the use of resignation as a coping style can adversely affect the patient as well as the clinical and social outcome of the illness.^17^ It would be worthwhile to examine cause–effect relationship between them. Possibly, a problem-solving approach by the caregiver could positively influence the patient's level of motivation leading to better socio-occupational functioning.

While this was a cross-sectional study, a prospective study would throw more light on the pattern of interaction between the factors studied. The caregivers were not screened for any psychopathology and there was a lack of literature for comparison with the study.

The implications of this study are that it will help us understand the burden of illness and coping styles used in India by caregivers of patients with a chronic mental illness such as schizophrenia, particularly in a non-institutionalized setting. Recent work at the University of California San Francisco (UCSF) Coping Project found that while caregivers do experience distress and depression, they also experience positive feelings. Family members often benefit from education about the illness, its treatment and family counselling that provides emotional support and practical advice on how to manage the patient's behaviour thereby minimizing the stress of caregiving.^31^

In the era of community mental health, it would help develop community-specific programmes to target caregivers for psychosocial intervention which would teach them to focus on the positive feelings they experience in association with the caregiving role and sustain this positive well-being with problem-focused mechanisms of coping. This could diminish the burden of illness felt by them, prevent ‘role overload’ and help them appreciate the ‘brief human moments’ that make caregiving worthwhile.
